# The mental representation of occupational stereotypes is driven as much by their affective as by their semantic content

**DOI:** 10.1186/s40359-022-00928-z

**Published:** 2022-09-21

**Authors:** Ferenc Kocsor, Tas Ferencz, Zsolt Kisander, Gitta Tizedes, Blanka Schaadt, Rita Kertész, Luca Kozma, Orsolya Vincze, András Láng

**Affiliations:** 1 Institute of Psychology, Faculty of Humanities and Social Sciences, University of Pécs, Pécs, Hungary; 2 Department of Behavioral Sciences, Medical School, University of Pécs, Pécs, Hungary

**Keywords:** Stereotypes, Representations, Person perception, Categorization

## Abstract

**Background:**

Studies on person perception showed that stereotypes can be activated by presenting either characteristic traits of group members, or labels associated to these groups. However, it is not clear whether these pieces of semantic information activate negative and positive stereotypes directly, or via an indirect cognitive pathway leading through brain regions responsible for affective responses. Our main objective with this study was to disentangle the effects of semantic and affective contents. To this end, we intended to scrutinize whether the representation of occupational labels is independent of the emotions they evoke.

**Methods:**

Participants (N = 73, M = 27.0, SD = 9.1, 31 men 42 women,) were asked to complete two tasks presented online. In the first task they had to arrange 20 occupational labels—randomly chosen from a pool of 60 items—in a two-dimensional space, moving the mouse pointer along two undefined axes. In a second task the axes’ names were defined a priori*.* Subjects were asked to arrange the labels according to *valence*, the extent to which the word evoked pleasant or unpleasant feelings, and *arousal*, the extent to which the word evoked excitement or calmness.

**Results:**

Based on the final coordinates of the labels, two cluster analyses were carried out separately in the two tasks. The two clusters were compared with Fisher’s exact test, which revealed that the cluster structures overlap significantly.

**Conclusions:**

The results suggest that the spontaneous categorization and the semantic representation of occupations rely largely on the affective state they evoke. We propose that affective content might have a primacy over detailed semantic information in many aspects of person perception, including social categorization.

**Supplementary Information:**

The online version contains supplementary material available at 10.1186/s40359-022-00928-z.

## Background

### Stereotypes evoked by occupational labels

The term *stereotype* refers to a generalized and simplified belief about a group of people [1], which forms through a socially embedded cognitive process that involves associating attributions to the group [2]. It is widely accepted that stereotypes might be formed based on direct observations [i.e. data-driven model of stereotypes, e.g., [3], or expectations held of a group [theory-driven model, e.g., [4], or reflect a combination of the two models [5]. It extends to physical appearance, interest, occupations or any similar characteristics held by a group of people [6–9]. Stereotypes as the contextual background of social perception result in that information about group members will be assimilated towards the stereotype, that is, they are being seen as more similar to the members of their own group [10]. Categorization is an inherent part of all perception [11] which also occurs after extremely brief exposure both in the visual [12] and the auditory modality [13]. In the social domain, peoples’ observable features evoke the process of instant categorization. Though categorization and the activation of stereotypes are distinct processes [14], stereotypes are activated automatically in most real-life and experimental settings as soon as someone is exposed to another individual [13–19]. A few attributes typical of a group are sufficient to infer group membership and behavioral characteristics [20]. For instance, clothing is used to infer other’s sexual preferences [21, 22] and to categorize them accordingly. When someone has been already categorized into a group, for example by their gender, it affects how their behavior is judged: people observing a man performing an act see him as more aggressive than a woman doing the same thing [20]. In addition to gender, age, ethnicity, beauty, and other stereotypes, occupational labels also activate automatic inference processes, and elicit the overall representation of a particular group [23].

While the content of stereotypes may differ between cultures, several principles of stereotyping are culturally universal [24, 25]. This includes the minimum requirement that is necessary for being able to talk about stereotyping, namely the phenomenon that all societies categorize and organize their members into subgroups [26]. The very basis of this is to classify people according to their age and gender. In societies in which resources are distributed unequally, more complex social constructs may divide the cultural groups further. This results in the divergence of groups based on financial resources, knowledge, and social relations in basically all Western and non-Western societies beyond the level of mere subsistence [25]. In countries with similarly structured societies the segmentation to subgroups has a lot in common. In a study conducted in the USA and Germany, participants were asked to form groups of listed professions [27]. A striking convergence was found between the stereotype dimensions in the categorization in the two countries. Participants of the study predominantly discriminated occupations based on agency and progressiveness, and to some extent on sociability as well. Further research discovered that the employees were not only identified by their job title, but assumed personality traits were also assigned to them based on their professions. It was also noticed that positive or negative perceptions of occupations in the same group were “transferred” to the rest of the group, so that the groups also received a kind of shared rating [27]. The conclusion is the same as in most previous studies on stereotypes [21–23]: if we do not have enough information about a person as an individual, inference will be based on categorical attributes.

### Semantic processing in person perception

One of the most systematic studies to date, which aimed to map how semantic information, delivered by occupational labels, is represented, was conducted by Imhoff et al. [27]. As noted in the previous section, their research focused on those possible dimensions that could serve as anchors when evaluating people. Undeniably, the number of dimensions and variations of spontaneous categorization could be countless. Therefore, the study focused on filtering out the most typical and practical ones. To obtain truly spontaneous stereotypes that best reflect what people usually think of first, the participants were free to come up with dimensions based on their own logic, and to classify and categorize the target labels according to these dimensions. The researchers found that the most frequent dimension by choice was *agency*. For instance, surgeons, software developers, and aerospace engineers were placed at the top of this dimension; cashiers, telemarketers, and parking attendants at the bottom. This suggests that agency refers to being powerful, assertive, and high in status. The second most frequent defining dimension was *progressiveness*. On this dimension, paramedics, firefighters, and police officers were labelled as conservative, conventional, and preventive (rule-based) types, while musicians, athletes, and designers were more likely to be labelled as liberal, alternative, and promotional types (characterized by innovation, risk-taking, brainstorming, etc.) [27, 28].

One obvious result of the study above is that it has shown that different occupations evoke similar stereotypes quite consistently. Certain social expectations may come with these resulting dimensions, which in turn can also be associated with evaluative judgements. Agency and progressiveness, beside providing superordinate categories for the occupations, deliver an affective meaning as well. Of course, people have idiosyncratic differences in how they relate to progressiveness; this term can have a positive meaning for some and negative for others. Agency, that is, having the knowledge and capability to deal with demanding situations, in contrast, is a term universally evaluated as a desirable feature (see 33). However, beyond the fact that some abstract categorization happens, we do not know much about the basic cognitive processes underlying these attributions.

Much of what we know about the cognitive processes of person perception comes from studies on the cognitive background of face perception. Bruce and Young's [29] cognitive model, later improved by Breen et al. [30, 31], shows that a key element of intact, conscious recognition of persons is that faces should also recall semantic information. These include names, occupations, places of residence, and so on. The so-called *person identification node* integrates visual information from the face with knowledge stored in memory. At the same time, characteristics that are not very specific to the individual—but are nevertheless important attributes, such as the aforementioned dimensions of agency, progressiveness, and sociability—might be parts of the features that are also involved in person recognition, just like they are elements of semantic information processing. Although the latter has not been investigated directly, there have been several concordant studies that show that facial appearance is used for spontaneous inference about trustworthiness and competence within a short time [32–35].

### Affective processing during face encoding—a model for understanding abstract representations

Just as stereotypes are based on observed behaviour and typical physical appearance, stereotypical descriptions activate representations of both expected behaviour and physical appearance. Therefore, we need to examine at which level evaluation of individuals happens in the first place: is it appearance, behaviour, or semantic knowledge? Each of these evoke affections directly, and the provided emotional content in turn contributes to the conscious recognition of persons, as it has been implied by the most influential face perception models [30, 36, 37]. Face recognition and face categorization are topics that have been studied extensively, therefore, theories of the cognitive processes in their backgrounds received a significant amount of empirical support. Face categorization is a special type of categorization which is, in real-life settings, usually involved in forming stereotypes and making social decisions. Hence, person perception cannot be understood in depth without having sufficient knowledge about face perception itself. Therefore, we will use face perception models as the starting point from which we intend to construct models that work on a more general representational level. To do this, it is necessary to review basic insights from said field, and highlight those cognitive and neural processes which might explain features of categorization in a more general sense, and those which might be paralleled with representation of semantic and affective contents.

The concept of affective space is anchored to face recognition models [7, 8], and is also present in research on person perception [38, 39]. Affective space is considered as a two-dimensional categorization system, where faces are assigned a place based on the emotions they evoke in the perceiver [40]. The dimensions of affective space are arousal (intensity) and valence (pleasantness). These dimensions can be thought of as the X and Y axes of a coordinate system where each face has a valence-arousal coordinate. In the dimensional approach, the intensity of the emotions we experience ranges, along the vertical axis, from low activation to high alertness (i.e., from calm to agitated, or bored to tense), while on the horizontal axis of the dimension the valence ranges from negative to positive (i.e., unpleasant-pleasant, sad-satisfied, upset-joyful). Valence refers to a kind of evaluation, or value attribution, that is subjectively induced by the appearance of emotions, while arousal refers to the level of activation or energization associated with emotions and their physiological characteristics [41, 42]. The study of Lang [41] explains emotional valence and arousal in terms of the functioning of specific motivational systems of the brain. According to the dimensional approach, the neural functioning of the two is mainly determined by valence, i.e., the emotional evaluation itself [42]. Valence is associated with the functioning of the two types of motivational systems in the brain and therefore plays a primary role [41]. According to Bradley and colleagues [40], the affective space can be equated with the approach-avoidance system, i.e. the appetitive and the aversive systems. The appetitive system is associated with pleasant things (exploratory behavior, eating, sexual behavior), but their intensity can vary from a relaxed state to an aroused state; while the aversive system deals with unpleasant consequences (avoidance, defensive behavior), also showing a large variance along the arousal dimension. The latter, since it communicates only differences in activation to the appetitive, the aversive, or both systems, has only a secondary, complementary role in the dimensional ordering of emotions [41].

The affective content elicited by unfamiliar faces depends primarily on the structural features of the face, its attractiveness, and how it is categorized by the observer. A divergence between affective processing and other processes involved in social cognition can be observed here as well: studies by Harris and Fiske [43, 44] revealed that faces implying having low competence and low warmth elicit responses in the amygdala and insula, that is, in regions playing a role in the processing of negative emotions, such as disgust, but no activation was measured in the medial prefrontal cortex, an area essential in social cognition. In person perception research, prejudices are typical cases of the expression of emotions. For someone who is prejudiced against a race, the perception of characteristic physical traits of that race is accompanied by the affective content of the prejudice [45]. The emotions evoked by the face of personal acquaintances and famous people, in contrast, depend on the specific experiences associated with that individual [46–49]. According to cognitive models of face perception, affective content plays an essential role in the conscious recognition of persons, in addition to semantic content. If the affective processing pathway is impaired, it leads to severe face recognition deficits. For instance, patients with Capgras-syndrome are able to recognize people who they have met before, however, the impairment of the so-called covert pathway of face recognition—which is responsible for affective contents—prohibits the acceptance of the fact that the observed faces are identical to those who are personally familiar [30, 36]. In contrast, patients suffering from prosopagnosia (i.e., an inability to consciously recognize familiar faces) show elevated physiological arousal (including increased heart rate and galvanic skin response) when they are exposed to faces of close acquaintances. In these individuals the overt pathway of face recognition is impaired, whereas the covert pathway is intact [30, 31, 50]. In summary, cognitive models of face recognition suggest that a sufficient affective charge is necessary for the activation of faces stored in the memory. Similarly, when approaching the process of face recognition from a representational view, we can say that stimulation of the affective space activates the associated region of the face space. Such affective content can be conveyed by labels that are known to activate stereotypes, that is, which evoke our expectations of behavior and appearance.

### Interconnectedness of representational spaces

The process of face recognition combines semantic and affective information to allow the recall of familiar faces. Above, we briefly summarized how this happens on the cognitive level. However, relatively little research has explored the interconnectedness of representational spaces, or tried to integrate the available pieces of information into a cognitive model. One notable attempt, called the Trait Inference Mapping (TIM) model [51], aimed to combine the concepts of face space with trait space.

However, this model has a rather monolithic view of trait space, and does not differentiate between the semantic and the affective contents that constitute the representation of traits. Furthermore, it only focuses on the physical aspect of people, namely faces. Nevertheless, the presentation of a face leads very rapidly to categorization, which in turn activates stereotypes related to that category. Hence, TIM is a good example of how different representational spaces might interact. Taking advantage of the approach of TIM, we aim to extend and generalize this model by suggesting that any aspect of a person—including facial appearance, group belonging, typical behavior, occupation, or any other characteristics—can be treated as an individual representational space, and thus be the subject of analysis. Similarly, trait space might be divided into semantic and affective spaces, and the latter one, if necessary, broken down to indices of valence and arousal. This is a similar approach to that utilized earlier by Stephan and Stephan [52]. They described stereotype activation as an interplay between cognition and affect (see also [53]). The terms they used, however, differ from those in the current manuscript. Cognitive processing in their wording is similar to what we call semantic representation; activations of affective states is similar to valence in our approach. Despite these differences, the inferences which can be drawn from their model can be paralleled with our expectations: when someone is exposed to a label describing a group, an emotional state (which involves certain levels of the feeling of pleasantness and activation as well) is elicited. It is important to note that this approach of differentiating between semantic information, and valence and arousal, serves the aims of a theoretical investigation; it is an abstract, purely cognitive model, and might not persevere when its neuroanatomical implications are tested.

### Aims and hypotheses

Person perception can be understood as a process of integrating different representational spaces. In this process, the image of each person is composed of elements that sometimes complement each other and sometimes mutually determine each other. Examples of such elements are *position in face space*, i.e. physical appearance (including facial symmetry, masculinity/femininity, skin texture, etc.), *semantically interpretable attributes* (group category, occupation, etc.), and *position in affective space*, determined by valence and arousal. These components may be interpreted at different levels of neural processing, but they can nevertheless be incorporated into a common cognitive model. In our first attempt to build a usable model of person perception, we focus on the processing of semantic and affective information. Therefore, the fundamental question of our research was to explore how big of a role the semantic content of labels, as well as the emotional responses they elicit, play in the processing of group-typical labels.

To this end, we designed an experiment where participants had to arrange occupational labels based on their semantic and their affective contents, respectively. Our analysis plan was to run cluster analyses on the arrangements to see whether the labels intuitively grouped together in the first task would show the same pattern in the second, formally instructed task as well. This analysis would reveal some of the connections between the two representational spaces. We expected that the representational space of affective contents would show a significant overlap with the space of semantic representations.

## Methods

### Participants

Our online study consisted of two tasks. Seventy-three people from Hungary participated in the first task (M = 27.0; SD = 9.1, min = 18; max = 58): 31 men (M = 27.9; SD = 10.3, min = 18; max = 56) and 42 women (M = 26,4; SD = 8,3; min = 18; max = 58). From this sample, 51 people completed the second part as well (M = 25.6; SD = 8.6, min = 18; max = 58): 24 men (M = 26.8; SD = 9.4, min = 20; max = 56) and 27 women (M = 24.6; SD = 7.8; min = 18; max = 58). Demographic data were analyzed using Jamovi 1.6.23 [43, 44].

### Procedure

We selected 53 occupational labels from the pool used by Imhof and colleagues [27], and translated these into Hungarian, the native language of the participants. Seven additional labels, which referred to criminal activity instead of occupations (e.g. drug dealer, mob member, thief, etc.), were added to the item pool in order to expand it with low valence labels (Additional file 1: Table S1). The two experiments were programmed in *Javascript*, and data were collected using *Qualtrics*.

In the first task, participants were asked to arrange 20 labels on the screen, using the *Spatial Arrangement Method* paradigm [28, 54]. The labels were randomly selected from the 60-item pool for each of the participants, and appeared on the screen, in a random arrangement, in four columns and five rows. The number of participants to whom a certain label was shown ranged between 16 and 33 (median = 25). After presenting the 20 labels together, participants were exposed to these consecutively one at a time during the task. They were instructed to arrange the labels on the screen with the mouse pointer, and use any aspects which they thought would adequately differentiate between labels. That is, occupational labels perceived as similar were more likely to be placed close to each other than labels evoking dissimilar concepts about the people they refer to. After completing the task, participants were also asked to report what name they would have given to the axes.

The second task was very similar to the first one, the only difference being that the participants had to arrange the labels along pre-specified dimensions. Specifically, it was explained that the X-axis refers to the extent to which each word evokes pleasant or unpleasant feelings (i.e., *valence*), and the Y-axis represents the extent to which the word evokes excitement or calmness (i.e., *arousal*). The endpoints of the axes were marked with adjectives that described the evoked states, such as sadness, anger, fear, depression (far left), happiness, good feeling, contentedness, hope (far right), excitement, awareness, enhanced attention (top), and calmness, boredom, and sleepiness (bottom), respectively. Hence, for example, labels that evoked positive feelings and excitement were expected to be clustered together in the top right-hand quarter of the screen. Similar to the first task, labels were selected randomly for each participant. The number of participants to whom a certain label was shown ranged between 11 and 25 (median = 17).

### Data processing

Based on the coordinates of the labels in the individual responses, the relative *semantic distances* between the occupations were calculated, using *R 4.1.0 *[55], with the following formula:

Relative distance (label_1; label_2) = (sqrt (((label_1_x – label_2_x)^2) + (label_1_y – label_2_y)^2)) / maxdist,

where label_1 and label_2 are the occupations whose distance are being calculated, label_1_x, label_1_y, label_2_x, and label_2_y are the X and Y coordinates of the labels, and maxdist is the maximal possible distance between two labels on the screen (i.e., the diagonal of the task area). After the standardized Euclidean distance of each pair had been calculated, each was averaged across all participants who had repositioned the two occupations. Then we transformed the relative distances into triangular form to allow multidimensional scaling with an ALSCAL procedure [56] using the *matrix* package in *R* [57]. It has been shown [27] that scaling stress fit is satisfying both in terms of scaling stress and parsimony for a 3 dimensional model. Therefore, we calculated the coordinates of each label for a 3D space using multidimensional scaling. Thus, we received three coordinates for each label. Coordinates collected in the second task were processed the same way: calculation of relative distances, averaging across participants, and multidimensional scaling.

Due to the fact that only a subset of labels was selected for each participant, the average relative distances between pairs differ in the number of observations their calculations were based on. From the possible 1770 combinations of labels each participant had to arrange only 20 pairs. This makes a total of 190 combinations for which a relative distance was collected from that particular person. In the free arrangement task, the number of participants who had to arrange a specific pair ranged between 2 and 18 (median = 8). In the affective task it ranged between 1 and 16 (median = 5). Hence, we had direct observational data for every single label combination. This range in the number of observations is quite high, and average relative distances between pairs that have been presented to participants infrequently may not represent the population average accurately. However, the multidimensional scaling relies on the average distances of all of the pairs, including the frequently presented as well, and tries to arrange the labels in a three dimensional space with the best fit. Therefore, the aforementioned differences in the number of observations between label pairs are unlikely to compromise the final results.

## Results

### Comparison of cluster content

We ran hierarchical cluster analyses on the coordinates of both the semantic and the affective spaces, respectively, with the Ward-method in *SPSS 26*. The visual inspection of the dendrograms suggested that participants positioned the labels into five distinct clusters in both spaces (Additional file 1: Figs. S1 and S2). For each semantic cluster we calculated the number of labels which have been positioned into that very cluster, while at the same time into one particular affective cluster. We did the same for each semantic-affective cluster combination. For instance, sailors and police officers were grouped in the first cluster in the affective space (A1) and the first cluster in the semantic space (S1), therefore the value of the respective cell (i.e., A1/S1) was 2. There was only one label (bookkeeper) in the fifth affective cluster (A5), which, at the same time, belonged to the fourth semantic cluster (S4). Hence, its cell count was 1 (Table 1).

**Table 1:** Number of labels belonging to each cluster pair

Cluster No.	Semantic clusters						Total
		S1	S2	S3	S4	S5	
Affective clusters	A1	2	0	9	7	0	18
	A2	0	2	0	0	5	7
	A3	4	0	3	8	0	15
	A4	0	0	2	0	0	2
	A5	9	0	8	1	0	18
Total	15	2	22	16	5	60	

To reveal whether the distribution of the affective clusters’ contents are independent from the distribution in the semantic space, we ran Fisher’s exact test using Monte Carlo simulation in *R* [55]. This test is a powerful alternative to the Chi-square for cases when its assumptions are not met. In this case the expected cell count is lower then 5 (60 labels distributed in 25 cells which gives on average 2.4). The accuracy of Fisher’s exact test is also greater on sparse tables which includes several zeros. For the calculation of the *p*-value all possible tables with matching margins would have to be generated which makes the Fisher’s exact test a rather memory intensive process. The Monte Carlo simulation serves to overcome the computational difficulties by randomly generating contingency tables with matching margins and assumes that for a sufficiently large number of runs, this will give a good estimation of the calculated tables.

If all values in Table 1 would be zeros, except one cell in each row and each column (for example the cells in the diagonal), it would mean that location of the occupational labels in the affective space determines their position in the semantic space as well. In contrast, if the numbers in the cells would be distributed evenly or arbitrarily, there would be a random overlap in the positioning of the labels in the two spaces. However, the simulated *p*-value of Fisher’s exact test, based on 1e + 07 replicates, showed that the arrangement of the labels in the two tasks was significantly different from a fully independent one (*p* = 0.0000001). The value of Cramer’s *V* was 0.57, which also confirmed that the two cluster analyses were similar to each other to a large extent.

### Categorization of axis labels

To receive an overview of the kind of representations the individual labels might have evoked in participants when they had a free choice to arrange them, the labels provided by the participants in the first task were grouped, categorized, and their frequency of occurrence analyzed (Table 2). Not all of the participants provided labels for both categories, and some of them added the same label to both axes. Therefore, cumulative label counts (n = 92) are lower than if everyone would have responded according to instructions. Though no clear correspondence could be detected, the received categories suggest that the 2D dimensions obtained with semantic arrangement align with those dimensions developed to characterize occupational roles by Imhoff et al. [27]. For instance, *agency* is one of the main factors, which is reflected in our study by the frequent use of axis names that refer to *knowledge*, *physical/intellectual work*, and *income*. Labels categorized along the *interesting/attractive* occupational axis might carry similar meaning as the dimension of *progressiveness*. Another dimension, *sociability*, appears in our responses as axis types such as *useful for the society* and *social desirability*.

**Table 2:** Frequencies of given axis names within label categories

Label categories	Counts	% of Total	Cumulative (%)
Knowledge	17	18%	18
Useful for the society	15	16%	35
Physical/intellectual work	12	13%	48
Income	15	16%	64
Social desirability	15	16%	80
Interesting/attractive	12	13%	93
Other	6	7%	100

## Discussion

In the present study, we aimed to investigate the representational level at which stereotypes are formed about people. More specifically, whether the semantic content of stereotypes, which can be activated by labels (i.e., the references to the person's appearance and behavioral characteristics), is an additional required factor in the categorization process, or whether the affective information evoked is sufficient, on its own, for categorization. To do so, participants had to arrange labels describing occupations in a two-dimensional space, first in a completely free semantic space, and then according to the elicited valence and arousal, that is, in the affective space. Since stereotype-activating labels will elicit some kind of affective response in any case, we expected that there would be an overlap between the two types of grouping. However, it is difficult to quantify the extent of the overlap. The only conclusion that can be drawn from the results of the analyses is that the groupings are not independent of each other. This cannot be attributed to the fact that the same participants were involved in both tasks, as the 20 labels to be clustered in each task were mostly different due to random selection from a 60-item pool. Nevertheless, the results show that participants in the experiment grouped the labels similarly in both situations. This suggests that the elicited emotional response already marks the place of the labeled occupations in the representational space. The additional information carried by the labels that more closely describes the behavioral patterns we can expect from people in the categories that the labels designate, does not in essence modify the representational map.

The results obtained are consistent with the predictions of theories that attempt to account for the formation of stereotypes associated with individuals at the level of representational spaces, but also go beyond to further clarify the cognitive processes that govern this. Over and Cook’s TIM model [51], for example, conceptualizes the process of categorization as a mapping between regions of face space and regions of trait space. Similarly, Kocsor and Bereczkei [7] found that, under experimental conditions, short behavioral descriptions assigned to individual faces can be used to shape the information stored about trustworthy and untrustworthy faces. Similar results were obtained with purely affectively loaded images that were socially irrelevant and uninformative in terms of semantic content [8]. This implies that both semantic and affective content can be generalized and they underlie an essentially affect-based decision. These findings are in compliance with the view we suggested in the introduction (2.4) that processing of information about people happens by mapping elements of numerous representational spaces onto each other: representation of physical appearance, behavioral characteristics, semantic knowledge, and affective information, all of which can be further broken down into overlapping layers of representations.

An analysis of the axes names given by the participants in the first task (Table 2) shows that the considerations in semantic clustering are mostly similar to those obtained in previous research [27]. Agency, progressiveness and sociability seem to be dominant aspects when it comes to typifying people and their associated occupational labels. The clusters created under these axes names were not different from those created under valence and arousal. Social utility, social desirability, and appeal have an obvious affective content. This is not the case for expertise, the time and effort required to acquire knowledge, or the physical or mental nature of the work. Nevertheless, the overlap of the clusters that emerge from the two different approaches shows that these contents, eventually, involve similar affective distinctions as the representations based on the valence and arousal induced by the labels. The semantic dimensions used for sorting are largely determined by the underlying affective content of the labels.

As noted above, the differences between the two clusters are difficult to quantify, so in this exploratory study we have merely sought to show that, although the categorization of individuals may be based on information of varying detail, the level of affective representation has already induced a sufficient level of stereotypical categorization. As a future prospect, it would be worth testing empirically whether the overlap between semantic and affective clustering actually influences behavior. In their study, Hills et al. [23] found that when faces are associated with labels that do not match their meaning, the time required for recognition is lengthened. Our current study implies that if discrimination based on meaning is essentially identical to discrimination based on elicited emotion, then we would expect equivalent results in a similar reaction time measurement task. That is, we can expect that incongruent priming stimuli (i.e., false labels) will produce roughly similar differences in face recognition speed, regardless of whether they differ semantically or affectively from what is stereotypically expected. If this is not the case, and semantic differences indeed cause a stronger increase in reaction times, then we need to reevaluate our results above and refine our model of person representation.

The latter is necessary in order to reconcile the theoretical approach of this study with the neuroanatomical models of person perception. The latter are mostly concerned with face recognition (e.g., [58, 59]), but also include the specification of the brain areas involved in the categorization of people. However, in addition to further empirical work, a theoretical integration that combines social psychological theories describing social decisions, cognitive models of representations, and neuroanatomical knowledge of person recognition and categorization is certainly needed. Although our present study is far from being a decisive step in this process, we hope that it will help pinpoint the cornerstones of a more accurate theoretical approach of person perception.

## Supplementary Information

**Additional file 1.** **Appendix. Table App1.** English translation of the list of occupational labels used in the experiment. **Figure App1.** Dendogram of the hierarchical cluster analysis which was run on the coordinates of the labels arranged freely in the semantic space. **Figure App2.** Dendogram of the hierarchical cluster analysis which was run on the coordinates of the labels arranged according to valence and arousal in the affective space.

## Data Availability

The datasets for this study, including SPSS data table and output file can be found in the Open Science Framework repository (https://osf.io/2d7t9/).

## References

[1] Allport GW (1954). The nature of prejudice.

[2] Semin GR. Stereotypes in the Wild. In: Stereotype Dynamics 2007 Aug 30 (pp. 22-39). Psychology Press.

[3] Hamilton DL, Gifford RK (1976). Illusory correlation in interpersonal perception: a cognitive basis of stereotypic judgments. J Exp Soc Psychol.

[4] Hamilton DL, Rose TL (1980). Illusory correlation and the maintenance of stereotypic beliefs. J Pers Soc Psychol.

[5] Haslam SA, Oakes PJ, Reynolds KJ, Turner JC (1999). Social identity salience and the emergence of stereotype consensus. Pers Soc Psychol Bull.

[6] Hilton JL, von Hippel W (1996). Stereotypes. Annu Rev Psychol.

[7] Kocsor F, Bereczkei T (2017). First impressions of strangers rely on generalization of behavioral traits associated with previously seen facial features. Curr Psychol.

[8] Kocsor F, Bereczkei T (2017). Evaluative conditioning leads to differences in the social evaluation of prototypical faces. Personal Individual Diff.

[9] McGarty C, Yzerbyt VY, Spears R (2002). Stereotypes as explanations: the formation of meaningful beliefs about social groups.

[10] Vonk R (2002). Effects of stereotypes on attitude inference: outgroups are black and white, ingroups are shaded. Br J Soc Psychol.

[11] Bruner JS (1957). On perceptual readiness. Psychol Rev.

[12] Mack ML, Palmeri TJ (2015). The dynamics of categorization: unraveling rapid categorization. J Exp Psychol Gen.

[13] Tsunada J, Cohen YE (2014). Neural mechanisms of auditory categorization: from across brain areas to within local microcircuits. Front Neurosci.

[14] Ito TA, Tomelleri S (2017). Seeing is not stereotyping: the functional independence of categorization and stereotype activation. Soc Cogn Affect Neurosci.

[15] Biernat M, Manis M, Nelson TE (1991). Stereotypes and standards of judgment. J Pers Soc Psychol.

[16] Bodenhausen GV, Wyer RS (1985). Effects of stereotypes in decision making and information-processing strategies. J Pers Soc Psychol.

[17] Duncan BL (1976). Differential social perception and attribution of intergroup violence: Testing the lower limits of stereotyping of Blacks. J Pers Soc Psychol.

[18] Kunda Z, Sherman-Williams B (1993). Stereotypes and the construal of individuating information. Pers Soc Psychol Bull.

[19] Sagar HA, Schofield JW (1980). Racial and behavioral cues in Black and White children’s perceptions of ambiguously aggressive acts. J Pers Soc Psychol.

[20] Futoran GC, Wyer RS (1986). The effects of traits and gender stereotypes on occupational suitability judgments and the recall of judgment-relevant information. J Exp Soc Psychol.

[21] Shelp SG (2003). Gaydar. J Homosex.

[22] Matthews C, Hill C (2011). Gay until proven straight: exploring perceptions of male interior designers from male practitioner and student perspectives. J Inter Des.

[23] Hills PJ, Lewis MB, Honey RC (2008). Stereotype priming in face recognition: Interactions between semantic and visual information in face encoding. Cognition.

[24] Cuddy AJC, Fiske ST, Kwan VSY, Glick P, Demoulin S, Leyens JP (2009). Stereotype content model across cultures: towards universal similarities and some differences. Br J Soc Psychol.

[25] Williams MJ, Spencer-Rodgers J (2010). Culture and stereotyping processes: integration and new directions. Soc Personal Psychol Compass.

[26] Sidanius J, Pratto F (1999). Social dominance: An intergroup theory of social hierarchy and oppression.

[27] Imhoff R, Koch A, Flade F (2018). (Pre)occupations: a data-driven model of jobs and its consequences for categorization and evaluation. J Exp Soc Psychol.

[28] Koch A, Imhoff R, Dotsch R, Unkelbach C, Alves H (2016). The ABC of stereotypes about groups: agency/socioeconomic success, conservative–progressive beliefs, and communion. J Pers Soc Psychol.

[29] Bruce V, Young AW (1986). Understanding face recognition. Br J Psychol.

[30] Breen N, Caine D, Coltheart M (2000). Models of face recognition and delusional misidentification: a critical review. Cogn Neuropsychol.

[31] Breen N, Coltheart M, Caine D (2001). A two-way window on face recognition. Trends Cogn Sci.

[32] Engell AD, Haxby JV, Todorov A (2007). Implicit trustworthiness decisions: automatic coding of face properties in the human amygdala. J Cogn Neurosci.

[33] Todorov A, Gobbini MI, Evans KK, Haxby JV (2007). Spontaneous retrieval of affective person knowledge in face perception. Neuropsychologia.

[34] Todorov A, Pakrashi M, Oosterhof NN (2009). Evaluating faces on trustworthiness after minimal time exposure. Soc Cogn.

[35] Willis J, Todorov A (2006). First Impressions making up your mind after a 100-ms exposure to a face. Psychol Sci.

[36] Ellis HD, Lewis MB (2001). Capgras delusion: a window on face recognition. Trends Cogn Sci.

[37] Ellis HD, Young AW (1990). Accounting for delusional misidentifications. Br J Psychiatry.

[38] Edwards K, von Hippel W (1995). Hearts and minds: the priority of affective versus cognitive factors in person perception. Pers Soc Psychol Bull.

[39] Pacilli MG, Mucchi-Faina A, Pagliaro S, Mirisola A, Alparone FR (2013). When affective (but not cognitive) ambivalence predicts discrimination toward a minority group. J Soc Psychol.

[40] Bradley MM, Codispoti M, Cuthbert BN, Lang PJ (2001). Emotion and motivation I: Defensive and appetitive reactions in picture processing. Emotion.

[41] Lang PJ (1995). The emotion probe: studies of motivation and attention. Am Psychol.

[42] Russell JA (1980). A circumplex model of affect. J Pers Soc Psychol.

[43] Harris LT, Fiske ST (2006). Dehumanizing the lowest of the low: neuroimaging responses to extreme out-groups. Psychol Sci.

[44] Harris LT, Fiske ST (2007). Social groups that elicit disgust are differentially processed in mPFC. Soc Cogn Affect Neurosci.

[45] Stangor C, Sullivan LA, Ford TE (1991). Affective and cognitive determinants of prejudice. Soc Cogn.

[46] Abelson RP, Kinder DR, Peters MD, Fiske ST (1982). Affective and semantic components in political person perception. J Pers Soc Psychol.

[47] Başar E, Schmiedt-Fehr C, Öniz A, Başar-Eroğlu C (2008). Brain oscillations evoked by the face of a loved person. Brain Res.

[48] Denkova E, Botzung A, Scheiber C, Manning L (2006). Implicit emotion during recollection of past events: a nonverbal fMRI study. Brain Res.

[49] Guerra P, Vico C, Campagnoli R, Sánchez A, Anllo-Vento L, Vila J (2012). Affective processing of loved familiar faces: integrating central and peripheral electrophysiological measures. Int J Psychophysiol.

[50] Bate S, Cook SJ (2012). Covert recognition relies on affective valence in developmental prosopagnosia: evidence from the skin conductance response. Neuropsychology.

[51] Over H, Cook R (2018). Where do spontaneous first impressions of faces come from?. Cognition.

[52] Stephan WG, Stephan CW. Cognition and affect in stereotyping: Parallel interactive networks. In: Affect, cognition and stereotyping 1993 (pp. 111-136). Academic Press.

[53] Ajzen I (2001). Nature and operation of attitudes. Annu Rev Psychol.

[54] Koch A, Alves H, Krüger T, Unkelbach C (2016). A general valence asymmetry in similarity: good is more alike than bad. J Exp Psychol Learn Mem Cogn.

[55] R Core Team. R: A Language and environment for statistical computing. [Internet]. R Foundation for Statistical Computing, Vienna, Austria; 2021. Available from: https://www.R-project.org/

[56] Young FW, Takane Y, Lewyckyj R (1978). ALSCAL: a nonmetric multidimensional scaling program with several individual-differences options. Behav Res Methods Instrum.

[57] Bates D, Maechler M. Matrix: Sparse and Dense Matrix Classes and Methods. R package version. 2021. Available from: https://CRAN.R-project.org/package=Matrix

[58] Liberman Z, Woodward AL, Kinzler KD (2017). The origins of social categorization. Trends Cogn Sci.

[59] Shutts K, Pemberton CK, Roben ES (2013). Children’s use of social categories in thinking about people and social relationships. J Cogn Dev.

