# Supplementary material for: The mental representation of occupational stereotypes is driven as much by their affective as by their semantic content
Source: BMC Psychol. 2022 Sep 21;10:222. doi: 10.1186/s40359-022-00928-z (PMC9494850; doi:10.1186/s40359-022-00928-z)
Supplement: Supplementary file 1 — Additional file 1. Appendix. Table App1. English translation of the list of occupational labels used in the experiment. Figure App1. Dendogram of the hierarchical cluster analysis which was run on the coordinates of the labels arranged freely in the semantic space. Figure App2. Dendogram of the hierarchical cluster analysis which was run on the coordinates of the labels arranged according to valence and arousal in the affective space. [file 40359_2022_928_MOESM1_ESM.docx]

**Appendix**

**Table App1.** English translation of the list of occupational labels used in the experiment

|  | Occupation |
| --- | --- |
| 1 | Managers |
| 2 | Lawyers |
| 3 | Psychologists |
| 4 | Judges |
| 5 | Aircraft Pilots |
| 6 | Programmers |
| 7 | Engineers |
| 8 | Criminal Investigators |
| 9 | Dentists |
| 10 | Doctors |
| 11 | Physicists |
| 12 | Veterinarians |
| 13 | Kindergarten Teachers |
| 14 | Teachers |
| 15 | Journalists |
| 16 | Fine artists |
| 17 | Bookkeeper |
| 18 | Meter Readers |
| 19 | Survey Researchers |
| 20 | Fishers |
| 21 | Telephone Operators |
| 22 | Highway Maintenance Workers |
| 23 | Farmworkers |
| 24 | Food Preparation Workers |
| 25 | Receptionists |
| 26 | Customer Service Representatives |
| 27 | Postal Service Workers |
| 28 | Waiters |
| 29 | Cleaning Workers |
| 30 | Barbers |
| 31 | Bartenders |
| 32 | Massage Therapists |
| 33 | Plumbers |
| 34 | Cooks |
| 35 | Car Mechanics |
| 36 | Embalmers |
| 37 | Explosives Workers |
| 38 | Athletes |
| 39 | Firefighters |
| 40 | Cashiers |
| 41 | Bus Drivers |
| 42 | Office Clerks |
| 43 | Clergy |
| 44 | Insurance Sales Agents |
| 45 | Police Officers |
| 46 | Musicians |
| 47 | Painters |
| 48 | Tailors |
| 49 | Secretaries |
| 50 | Sailors |
| 51 | Nurses |
| 52 | Parking Lot Attendants |
| 53 | Chief Executives |
| 54 | Mob member |
| 55 | Usurers |
| 56 | Thieves |
| 57 | Drug Traffickers |
| 58 | Fences |
| 59 | Bank Robbers |
| 60 | Peculators |

**Figure App1.** Dendogram of the hierarchical cluster analysis which was run on the coordinates of the labels arranged freely in the semantic space


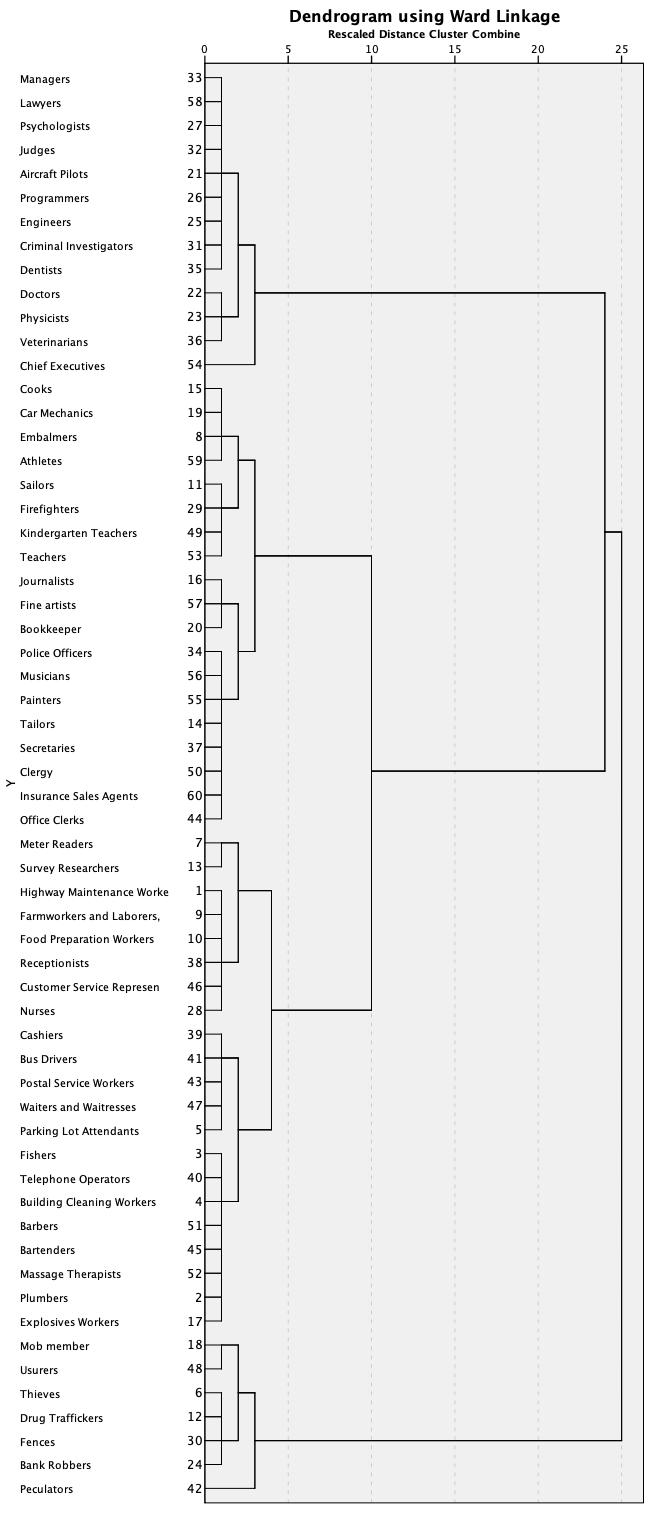


**Figure App2**.
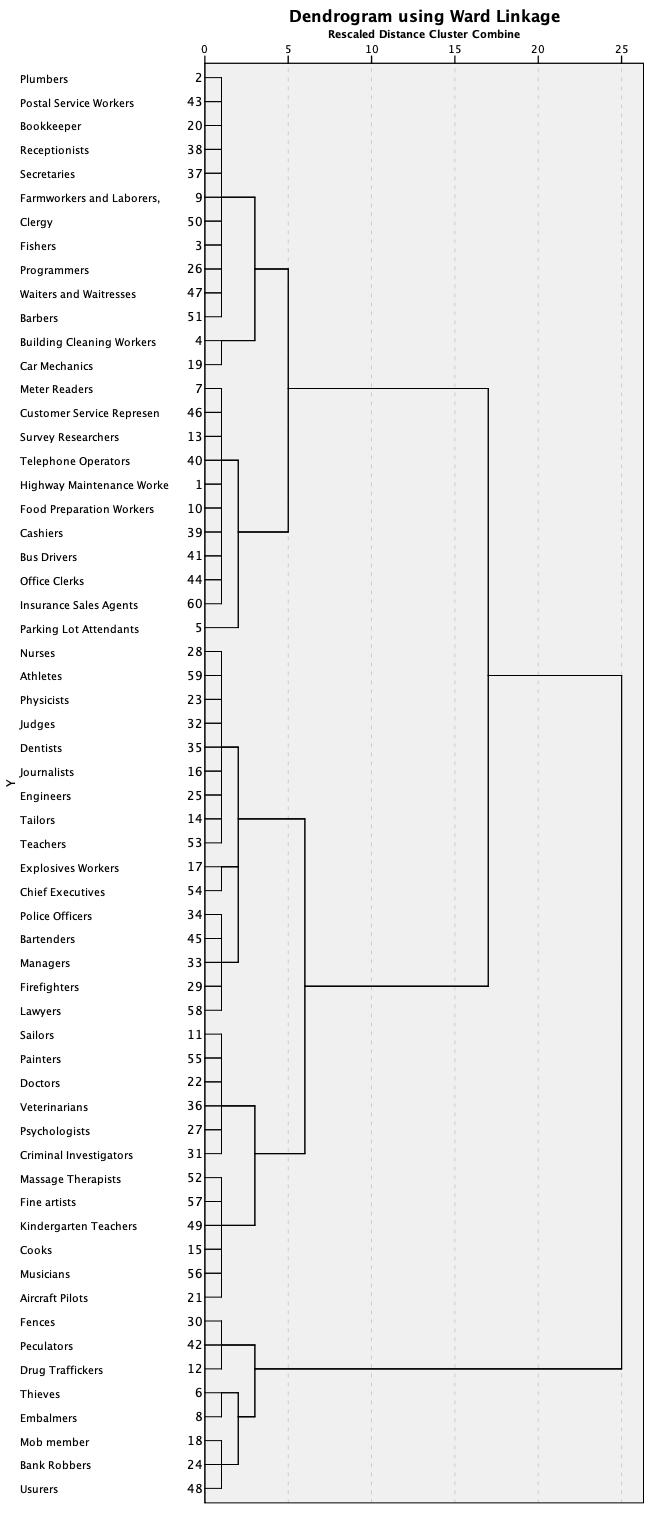
Dendogram of the hierarchical cluster analysis which was run on the coordinates of the labels arranged according to valence and arousal in the affective space
